# Design and Protocol of a Randomised Controlled Trial Evaluating Virtual Reality to Improve Patient Experience During PICC and PICC-PORT Placement in Oncology Patients

**DOI:** 10.3390/nursrep16050165

**Published:** 2026-05-13

**Authors:** Carlo Alberto Camuccio, Paola Tiatto, Orejeta Diamanti, Elisabetta Bisinella, Rachele Loro, Alice Bernardi, Martina Berto, Federica Turchet, Andrea Rostirolla, Elena Reginato, Shabnam Zohrabi, Weisha Qi, Matteo Bernardi

**Affiliations:** 1 Immunology and Molecular Oncology Diagnostics Unit, Veneto Institute of Oncology IOV-IRCCS, Via Gattamelata 64, 35128 Padua, Italy; alberto.camuccio@iov.veneto.it (C.A.C.); shabnam.zohrabi@iov.veneto.it (S.Z.); weisha.qi@iov.veneto.it (W.Q.); 2 Unit of Anesthesia and Intensive Care 2, Veneto Institute of Oncology IOV-IRCCS, Via Dei Carpani 16/Z, 31033 Castelfranco Veneto, Italy; paola.tiatto@iov.veneto.it (P.T.); elisabetta.bisinella@iov.veneto.it (E.B.); rachele.loro@iov.veneto.it (R.L.); alice.bernardi@iov.veneto.it (A.B.); martina.berto@iov.veneto.it (M.B.); federica.turchet@iov.veneto.it (F.T.); andrea.rostirolla@iov.veneto.it (A.R.); 3 Scientific Direction, Veneto Institute of Oncology IOV-IRCCS, Piazza Salvemini 13, 35131 Padua, Italy; 4 Health Professions Directorate Unit, Veneto Institute of Oncology IOV-IRCCS, Via Gattamelata 64, 35128 Padua, Italy; elena.reginato@iov.veneto.it (E.R.); matteo.bernardi@iov.veneto.it (M.B.)

**Keywords:** virtual reality, neoplasm, PICC placement, vascular access devices, patient satisfaction, patient-relevant outcome, randomised controlled trial, study protocol, digital health, complementary therapies

## Abstract

**Background/Objectives**: The placement of central venous access devices, including peripherally inserted central catheters (PICCs) and PICC-PORTs, is a routine procedure in oncology care. Usually associated with limited physical pain, these procedures may nevertheless generate significant anxiety and negatively influence the overall procedural experience. Virtual reality (VR) has emerged as a non-pharmacological intervention capable of modulating attentional and emotional responses during medical procedures; however, evidence in adult oncology patients undergoing vascular access placement remains scarce. The aim of this study is to evaluate the effect of VR on an oncological patient’s overall procedural experience. **Methods**: This manuscript outlines the design and methodology of a prospective, single-centre randomised controlled trial. Adult oncology patients scheduled for PICC/PICC-PORT placement are randomised to receive standard care alone or standard care combined with an immersive VR intervention delivered via a head-mounted display during the procedure under pragmatic, real-world clinical conditions. The primary outcome is a composite patient-reported procedural experience endpoint, assessed through a non-aggregated framework encompassing procedural anxiety, comfort, satisfaction and procedural tolerability. Procedural anxiety constitutes the main quantitative driver; the remaining domains are analysed as individual component dimensions and interpreted jointly to contextualise the overall experience. Secondary outcomes include procedural pain, physiological parameters and procedural characteristics. A mixed-methods approach integrates quantitative assessment with qualitative phenomenological analysis. **Results**: The study is expected to provide methodological and clinical insight into the role of immersive VR in improving procedural experience and support future multicentre trials. **Conclusions**: This trial will contribute to the expanding field of digital and immersive health technologies by evaluating VR as a patient-centred adjunct intervention in oncological procedural care using a predefined patient-reported experience-based primary endpoint. The protocol has been submitted to ClinicalTrials.gov with the registration number NCT07384741.

## 1. Introduction

After recent advances in virtual reality (VR), this technology has been adopted in healthcare for a range of interventions, from clinical training and rehabilitation to mental health and pain management. VR systems offer an immersive and multisensory experience capable of capturing patients’ attention and potentially affecting their perception of medical procedures.

Recent studies have shown that, compared with conventional distraction techniques, VR has proven more successful in modulating pain and anxiety during clinical procedures by redirecting the patient’s focus towards engaging, calming environments and potentially influencing the neuromatrix of pain and anxiogenic input [1]. This process is thought to reduce the subject’s perception of discomfort and promote a state of relaxation and emotional containment [2,3,4]. Given that the side effects of virtual reality are generally mild, primarily including dizziness or nausea, often referred to as cybersickness [5,6], VR is considered a feasible and beneficial complement to standard care under local anaesthesia.

To date, the paediatric population has generated the most data supporting VR use in clinical settings [7], while adult patients undergoing invasive procedures are less studied. Nonetheless, recent studies in adult settings suggest that the use of VR may result in a reduction in procedural pain and anxiety without modifying or prolonging the duration of the procedure or increasing pharmacological sedation [3]. However, these studies are largely focused on the use of virtual reality in surgical settings; indeed, the recent meta-analysis by Lassen et al. [3] includes 37 articles, the majority of which were conducted in operative contexts, and only three investigate the use of VR during the insertion of central venous access devices (CVADs), which is the focus of the present protocol paper.

Oncology patients often require the placement of CVADs due to long-term intravenous treatments such as chemotherapy [8,9]. The placement of PICCs and PICC-PORTs is a minimally invasive procedure and does not result in immediate complications [10,11]; it is preferred because it avoids the use of peripheral venous access, which is unsuitable for most chemotherapeutic agents, reducing the risk of endothelial injury and extravasation of vesicant drugs, and allowing therapies to be delivered in outpatient or home-care settings [10]. However, despite these procedural and organisational advantages, patients may experience psychological distress associated with fear of the unknown, the emotional burden of a cancer diagnosis, and concerns about pain [12,13]. While procedural pain is usually described as mild and mainly related to the administration of local anaesthesia, anxiety levels appear variable and are strongly influenced by individual expectations and emotional vulnerability [14,15,16].

These observations indicate that CVAD insertion is not adequately described by pain intensity alone, but also by emotional and contextual components that play a role in the patient’s experience. This distinction is particularly crucial in oncological settings, where repeated and prolonged care pathways are common, and where patient experience and satisfaction can directly influence clinical outcomes and adherence to treatment [15].

As mentioned in the previous paragraphs, studies investigating the use of virtual reality during CVAD insertion are relatively limited: of the three randomised controlled trials included in the meta-analysis by Lassen et al. [3], two out of three use ports as the vascular access device [16,17], while only one focuses on PICC insertion [15]. These three studies confirm that the use of virtual reality appears to be a valid non-pharmacological strategy aimed at improving the overall procedural experience by reducing anxiety and pain scores without the need for increased analgesics or prolonged procedure times [16]. Virtual reality also appears to increase comfort, support a sense of emotional control, and enhance patient satisfaction and perceived quality of care [15,16,17], although only the study by Sargut et al. [16] reports statistically significant results.

Therefore, these studies conducted in the context of oncological CVAD placement support this broader clinical value in adult interventional procedures. Overall, while the aforementioned studies provide valuable preliminary evidence supporting feasibility and symptom modulation, they primarily focus on isolated symptom-based outcomes and do not evaluate procedural experience as a multidimensional patient-reported construct. In particular, no prior randomised controlled trial has been explicitly designed with patient-reported procedural experience as a predefined primary outcome in adult oncology patients undergoing PICC or PICC-PORT placement.

This study describes the design and methodology of a randomised controlled trial evaluating the effectiveness of an immersive VR intervention, delivered via a head-mounted display, during PICC and PICC-PORT placement in adult patients in an oncological setting.

### Outcomes and Aim

The primary outcome of this study is a composite patient-reported procedural experience endpoint, assessed immediately after the procedure. The primary analytical component of this composite endpoint is procedural anxiety, measured using a 10-point Visual Analogue Scale for Anxiety (VAS-A). The main statistical analysis will compare changes in VAS-A scores between the intervention group and the control group.

The additional dimensions of the composite endpoint, patient-reported comfort, satisfaction and procedural tolerability are collected concurrently and are not aggregated into a single score. These dimensions are analysed separately and interpreted jointly with procedural anxiety in order to provide an overall assessment of the patient’s procedural experience.

Secondary outcomes include: (1) procedural pain, assessed using a 10-point Visual Analogue Scale (VAS); (2) physiological parameters, including heart rate and arterial blood pressure; (3) procedural characteristics, such as procedure duration and number of device insertion attempts; (4) qualitative assessment of patients’ perceptions and lived experiences of the procedure, analysed using Van Kaam’s phenomenological method [18].

The aim of this study is to evaluate the effect of an immersive virtual reality intervention on the overall procedural experience of adult oncology patients undergoing PICC and PICC-PORT placement, with patient experience conceived as a primary dimension of quality of care.

## 2. Materials and Methods

### 2.1. Study Design

This study is designed as a prospective, interventional, randomised controlled trial (RCT) with a parallel-group design, conducted under real-world clinical conditions. The trial is conceived as an exploratory, pragmatic RCT aimed at evaluating the effectiveness of an immersive virtual reality (VR) intervention used as a non-pharmacological adjunct during PICC and PICC-PORT placement in adult oncology patients.

Participants are randomly allocated in a 1:1 ratio to one of two study arms: standard of care alone (SOC) or standard of care combined with immersive VR (VR + SOC). The study adopts a pragmatic approach, integrating the intervention into routine clinical practice without modifying standard procedural workflows and organisational conditions.

Due to the nature of the intervention, blinding of participants and healthcare professionals is not feasible. The trial is conducted at a single tertiary oncology centre and follows a superiority framework, with a composite patient-reported procedural experience endpoint as the primary outcome. This study protocol was developed in accordance with the Standard Protocol Items: Recommendations for Interventional Trials (SPIRIT) 2025 Statement [19].

The study is registered in ClinicalTrials.gov (ID: NCT07384741).

### 2.2. Study Setting

The study is conducted at a research-focused oncology hospital in Northern Italy; it is a tertiary referral cancer centre within clinical areas routinely dedicated to the placement of central venous access devices. All procedures are performed according to institutional protocols for PICC and PICC-PORT insertion and under standard clinical conditions, ensuring that the intervention is fully integrated into routine care workflows without introducing additional burden for patients or staff.

Patient recruitment, intervention delivery, and data collection take place in real-world clinical settings, without modifications to standard procedural pathways, thereby enhancing the external validity and applicability of the study findings to everyday oncological practice.

### 2.3. Participants

Patients and members of the public were not formally involved in the planning or development of this study. Their contribution was indirectly integrated through insights from the literature, observations from routine clinical practice and by actively listening to patients’ opinions prior to the study, which informed the research questions and the choice of patient experience as the primary outcome. Although patients did not directly contribute to the intervention or procedures, informal feedback supported the acceptability of wearing the VR headset. Upon publication, participants will receive a summary of the findings, which will also be disseminated through institutional channels and oncology patient organisations.

### 2.4. Eligibility Criteria

Patients are eligible for inclusion if they meet all of the following criteria:

- confirmed oncological diagnosis;
- scheduled placement of a PICC or PICC-PORT as part of routine clinical care;
- adequate understanding of the Italian language;
- ability to provide written informed consent.

Patients are excluded if one or more of the following conditions are present:

- refusal to participate or inability to provide informed consent;
- cognitive, psychiatric or neurological conditions that may compromise the reliability of patient-reported outcomes or the ability to follow the basic instructions related to the procedure;
- significant visual or auditory impairment preventing effective use of the VR headset;
- sensory deficits involving the anatomical area of device insertion.

Eligible patients are recruited consecutively and enrolled according to the centralised predefined randomisation sequence.

### 2.5. Randomization and Allocation

Participants are randomly assigned to one of the two study arms in a 1:1 allocation ratio. The randomisation sequence is generated using an independent, certified web-based randomisation service (Sealed Envelope™, London, UK). Simple randomisation is employed [20], without any a priori constraint on final group size, consistent with the pragmatic, real-world nature of the trial.

According to the pragmatic approach of the study, no stratification procedures are applied. Allocation concealment is maintained through centralised, real-time assignment by the online system. After eligibility is confirmed and informed consent obtained, the treating clinician accesses the secure Sealed Envelope web interface, which reveals the allocation at the point of assignment, thereby ensuring concealment at the time of assignment and preventing foreknowledge of upcoming allocations.

Given the characteristics of the intervention, blinding is not feasible at any stage of the study. Participants are aware of whether they are receiving the VR intervention, and healthcare professionals involved in device placement are likewise aware of group allocation.

Blinding of outcome assessment is not feasible for the primary and secondary patient-reported outcomes, which are collected directly from participants aware of their allocation.

All outcomes are collected using standardised instruments applied uniformly across study arms. To mitigate the risk of bias, the operator performing the procedure is not involved in the collection of patient-reported outcomes, and objective parameters (physiological and procedural data) are extracted from clinical records by research personnel not involved in the procedure. The absence of blinding is acknowledged as an inherent methodological limitation and is considered in the interpretation of study findings.

### 2.6. Intervention

#### 2.6.1. Virtual Reality Plus Standard of Care (Vr + Soc)

Participants allocated to the intervention group undergo PICC or PICC-PORT placement according to institutional standard procedures, combined with the use of an immersive virtual reality system delivered via a head-mounted display during the procedure.

The VR system used is a commercially available Class I non-sterile medical device, compliant with EU Regulation 2017/745 (Medical Device Regulation) [21]. The device delivers immersive audiovisual content, integrating visual environments, guided breathing, and structured audio designed to promote relaxation and attentional engagement. See Supplementary Materials File S1, for further technical information.

The VR device does not record, store or transmit any personal or clinical data, and its use does not involve data processing activities beyond content visualisation. Prior to the procedure, patients may select among different visual and audio environments. Throughout the intervention, verbal interaction with healthcare professionals is continuously maintained, assuring safety and preserving the usual communicative flow of the procedure.

The VR intervention is implemented exclusively as a supportive, non-pharmacological adjunct to standard care and does not replace clinical communication, procedural monitoring or operator–patient interaction during device placement. All standard clinical care procedures are permitted and are not altered by the intervention. In the case of cybersickness or clinically relevant symptoms such as nausea, dizziness or visual discomfort, the operator is ready to assess and intervene, including removing the VR device if necessary. In such cases, the procedure continues according to standard care.

#### 2.6.2. Standard of Care (Soc)

Participants allocated to the control group undergo PICC or PICC-PORT placement according to the same institutional protocols, without the use of VR. All clinical aspects of care, including local anaesthesia, monitoring and procedural management are identical to those applied in the intervention group.

#### 2.6.3. Standardisation and Expectancy Control

To minimise expectancy and placebo effects, a standardised information procedure is applied to both study groups. Information is delivered according to a predefined script and administered by a specifically trained registered nurse who is not involved in outcome assessment.

Participants allocated to the control group receive neutral standard information regarding the timing and phases of the procedure. Participants allocated to the VR group additionally receive a brief (approximately 120 words), neutral description of headset use, including instructions that they will wear the device during the procedure, that they may view different visual scenarios and instructions on how to promptly communicate any discomfort or issues to the clinical staff. This information explicitly excludes any reference to potential benefits or expected effects of VR use.

Operators are instructed not to provide feedback or engage in discussion about the content experienced during or immediately after the intervention in either group, in order to avoid differential attention and expectancy-related bias.

### 2.7. Outcomes

The primary outcome of the study is a composite patient-reported procedural experience endpoint, reflecting the overall effectiveness of the virtual reality intervention during PICC and PICC-PORT placement. Although the term “composite endpoint” is used, it refers here to a conceptual and experiential framework rather than to a statistically aggregated outcome.

This composite endpoint integrates multiple dimensions of procedural experience, including:

- change in procedural anxiety, which represents the prespecified principal quantitative driver of the endpoint;
- patient-reported comfort and satisfaction with the procedure;
- perceived procedural acceptability and overall tolerability.

Procedural experience is assessed immediately after completion of the procedure (T1) using structured patient-reported measures, combining ordinal rating scales and qualitative evaluation to assess complementary quantitative and experiential elements. Procedural anxiety (measured using the 10-point Visual Analogue Scale for Anxiety, VAS-A) constitutes the primary focus of analysis, while the remaining component dimensions (comfort, satisfaction and procedural tolerability) are analysed as individual, non-aggregated measures and interpreted jointly with anxiety to provide an overall understanding of procedural experience. No numerical aggregation of component scores is applied.

This approach reflects the multidimensional and subjective nature of immersive digital interventions, for which patient experience constitutes a clinically meaningful outcome.

Secondary outcomes include:

- procedural pain, assessed using a 10-point Visual Analogue Scale (VAS);
- physiological parameters, including heart rate and systolic, diastolic and mean arterial pressure as routinely monitored;
- procedural characteristics, including procedure duration and number of insertion attempts;
- qualitative assessment of patients’ perceptions and lived experiences related to the procedure, analysed using Van Kaam’s phenomenological method [18].

This exploratory analysis aims to elucidate the relationship between procedural anxiety and the other experiential dimensions and to facilitate comparison with previous studies in which anxiety was evaluated as a standalone outcome. Procedural pain and anxiety are therefore analysed separately to explore their individual contributions to the overall procedural experience.

### 2.8. Data Collection and Timing

Recruitment strategy

Participants are recruited consecutively from patients scheduled to undergo PICC or PICC-PORT placement at the hospital where the study is conducted. Screening for eligibility, based on the predefined inclusion and exclusion criteria, is performed on the day of the procedure by a member of the study nursing team. Eligible patients are then approached prior to entry into the procedural room or outpatient surgical area, provided with detailed study information and invited to participate. In the case of a positive response, written informed consent is obtained.

Based on institutional activity in 2024, with an average of approximately 15–20 PICC/PICC-PORT insertions per week, a recruitment rate of around 8–10 patients per week can be conservatively anticipated. Accordingly, the target sample size of 120 participants is expected to be reached within approximately 10–12 weeks of active, continuous recruitment. Participant flow through the study will be documented using a CONSORT-compliant flow diagram, anticipated to guide reporting of the main trial results [22].

**Figure 1:**
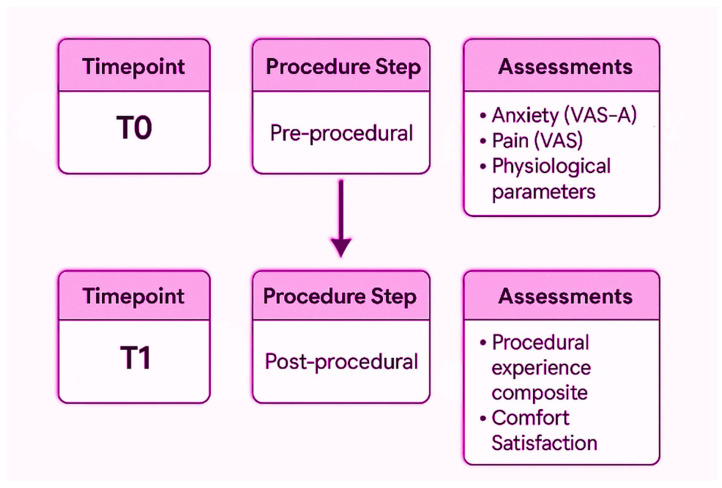
Participant timeline of assessments (T0 pre-procedure and T1 post-procedure), detailing variables, measurement tools, and time points for baseline and post-procedural data collection.

Data are collected at two predefined time points using a structured case report form specifically developed for the study and subsequently entered into the REDCap electronic database, Figure 1.

- T0 (pre-procedural): within 2 min of room entry and prior to preparation of the sterile field, baseline procedural anxiety, pain intensity and physiological parameters (heart rate and arterial blood pressure) are recorded.
- T1 (post-procedural): within 2 min of completion of the procedure, after removal of the VR headset (for the intervention group) and before the patient leaves the procedural room, patient-reported outcomes related to procedural experience and satisfaction are collected, together with brief qualitative questions exploring patients’ perceptions and experience of the procedure.

Data quality and qualitative rigour

Qualitative data are collected using open-ended prompts administered by a trained research nurse who is not involved in PICC/PICC-PORT insertion or intervention delivery.

The qualitative component follows Van Kaam’s phenomenological method [18] both in data collection and analysis. Methodological rigour is supported through: (a) interviewer training in neutral and non-directive questioning; (b) reflexive documentation by the research team; (c) maintenance of an audit trail; and (d) regular analytic oversight to ensure consistency and transparency of interpretation, in accordance with established qualitative research criteria [23].

As all assessments take place during a single clinical encounter, no specific retention strategies are planned. The only potential source of missing data is interruption of the procedure for clinical reasons (e.g., patient distress or technical failure), which will be documented and reported in the CONSORT flow diagram.

### 2.9. Measures

#### 2.9.1. Quantitative Measures

Anxiety and pain are assessed using validated 10-point visual analogue scales, respectively: VAS-A, with scores ranging from 0 (no anxiety) to 10 (worst possible anxiety), and a 10-point VAS for pain.

Physiological parameters, including heart rate and arterial blood pressure, are recorded through standard clinical monitoring devices routinely used during vascular access placement.

Local anaesthetic administration follows institutional protocols. The type and total administered dose of anaesthetic agent are recorded descriptively as part of the data collection.

Patient-reported comfort and procedural tolerability are measured using 5-point Likert scales (1 = very poor to 5 = excellent). Patient satisfaction is assessed using a 10-point Visual Analogue Scale ranging from 0 (not at all satisfied) to 10 (completely satisfied).

#### 2.9.2. Qualitative Component

Qualitative data are collected using open-ended prompts and analysed according to Van Kaam’s phenomenological method [18]. Procedures to ensure qualitative rigour and trustworthiness, including interviewer training, reflexive documentation, audit trail maintenance and analytic oversight, are described in Section 2.8.

See the Supplementary Materials to view the approved dataset, File S2.

### 2.10. Data Management Plan and Analysis

Study data will be entered into an electronic database in REDCap, used exclusively for data management and not for randomisation. All entered data will be pseudonymised, and access will be restricted to personnel specifically authorised for the study through dedicated password-protected accounts. The data will be stored on an institutional server of the study hospital and retained according to the relevant regulations. Identifiable information will be kept separate from research data, ensuring full confidentiality.

De-identified individual participant data, the data dictionary and the statistical code will be made available upon reasonable request to the Principal Investigator. No identifying information will be shared.

Given the minimal-risk nature of the study and its single-centre design, no formal monitoring of trial conduct is planned. The research team will oversee the study, protocol adherence and data collection as part of routine clinical practice.

All analyses will be conducted according to the intention-to-treat (ITT) principle. Descriptive statistics will be used to summarise participants’ demographic and clinical characteristics. Continuous variables will be reported as means and standard deviations or medians and interquartile ranges, as appropriate, according to variable distribution, while categorical variables will be presented as frequencies and percentages.

For the primary composite outcome, between-group comparisons will focus on procedural anxiety (VAS-A) as the prespecified principal quantitative driver of the composite endpoint. Independent samples Student’s *t*-tests, or non-parametric equivalents when appropriate, will be used for between-group analyses. Patient-reported comfort, satisfaction and procedural tolerability will be analysed as individual, non-aggregated component dimensions of the composite endpoint using appropriate parametric or non-parametric methods and interpreted jointly with anxiety. Secondary quantitative outcomes, including procedural pain and physiological parameters, will be analysed using similar inferential methods.

Within-group pre- and post-procedural comparisons will be explored using paired statistical tests. Statistical significance will be set at a two-sided *p* < 0.05.

The planned sample size is based on a power-based rationale focusing on procedural anxiety as the principal quantitative driver of the primary composite outcome. Assuming a clinically relevant between-group difference of approximately 1.5 points on a 10-point VAS-A, a standard deviation of 2.5, a two-sided alpha level of 0.05 and 80% power, a minimum of 44 participants per group is required. To account for uncertainty in effect size estimates, anticipated variability in a real-world clinical context and potential missing data or protocol deviations, the target sample size was increased conservatively to approximately 120 participants, consistent with the trial registration.

Missing data will be handled using complete-case analysis. If the proportion of missing data exceeds 10% for any key variable, sensitivity analyses may be performed to assess the robustness of the results.

No formal multivariable adjustment is planned, in line with the exploratory and pragmatic nature of the trial and the sample size, which is powered for the anxiety-driven component of the primary outcome. Potential confounders (e.g., baseline anxiety, pharmacological therapies and clinical context) are prospectively collected to support informed interpretation of outcome variability rather than formal statistical adjustment.

The study team will perform periodic internal monitoring of data completeness and protocol adherence using REDCap audit trails.

Qualitative data will be analysed using Van Kaam’s phenomenological method [18], involving systematic coding, identification of recurring themes and synthesis of the essential meanings of patients’ lived experiences. Quantitative and qualitative findings will be integrated at the interpretation stage using a narrative approach, whereby qualitative themes are used to contextualise and deepen understanding of the quantitative results. No formal mixed-methods integration techniques (e.g., data transformation or joint displays) are planned, given the protocol focus and exploratory aims of the qualitative component. Statistical analyses will be conducted using dedicated statistical software.

## 3. Ethical Considerations

The study is conducted in accordance with the principles of the Declaration of Helsinki. All participants provide written informed consent prior to enrolment, after receiving comprehensive information about the study from trained members of the study team, and are informed of their right to withdraw from the study at any time without consequences for their standard clinical care.

The study protocol, including all amended documents, has been reviewed and approved by the Comitato Etico Territoriale Area Nord Veneto (CET-ANV). Following submission and evaluation of the requested amendments, the Ethics Committee issued a final favourable opinion (internal code CET-ANV 2025-18, Version 1.0, dated 5 September 2025) authorising the conduct of the study.

Any protocol amendments will be communicated to the Ethics Committee and updated in the trial registry (ClinicalTrials.gov).

Study results will be disseminated through publication in peer-reviewed journals, presentations at scientific conferences and summary reports provided to participants upon request.

The virtual reality device complies with European medical device regulation and does not involve acquisition, storage or processing of patient data, therefore posing no additional risks in terms of data protection or privacy for study participants.

A Data Monitoring Committee (DMC) is not planned for this study, given the minimal risk of the intervention, which does not involve the use of drugs, and its complementary nature to routine clinical procedures. No additional risks beyond standard clinical procedures are anticipated.

Given the minimal risk of the intervention, no ancillary post-intervention care is planned. Participants will continue to receive standard care according to institutional clinical protocols.

## 4. Discussion

This manuscript describes the rationale and design of a randomized controlled trial investigating the use of immersive virtual reality (VR) during PICC and PICC-PORT placement in adult oncology patients. The study is grounded in the growing interest in digital and immersive technologies as non-pharmacological adjuncts aimed at improving patient-centred care, particularly in procedural contexts characterized by emotional vulnerability and anticipatory anxiety.

By adopting a composite patient-reported procedural experience endpoint as the primary outcome, this study contributes to addressing an existing gap in the literature, as, to our knowledge, no randomised controlled trials have evaluated the use of virtual reality during PICC and PICC-PORT placement using a qualitative and multidimensional, non-aggregated patient-reported framework in adult oncology patients.

CVADs are a routine procedure in the therapeutic pathway of oncology patients, often associated with low levels of physical distress, yet they frequently trigger significant emotional and psychological burdens. These feelings arise from transitions in the therapeutic pathway, fear of the unknown and the broader experience of a cancer diagnosis and treatment. In this context, approaches focusing exclusively on nociceptive stimuli may not fully capture the complexity of patients’ procedural experience. This study aims to address this by selecting a composite patient-reported procedural experience endpoint as the primary outcome. This paradigm differs from traditional symptom-based measures such as pain and anxiety scores. Immersive technologies like VR may prove more effective in shaping the emotional and experiential aspects of care, rather than focusing on reductions in clinical symptoms. Indeed, procedural anxiety is the principal quantitative driver for inferential analysis. Comfort, satisfaction, and procedural tolerability are analysed as individual component dimensions and interpreted jointly to contextualise the experiential impact of the intervention beyond anxiety reduction alone. This approach is consistent with our hypothesis that immersive VR exerts its effects not only through symptom modulation but also by shaping the emotional and experiential context of the procedure [15,16].

From a methodological perspective, the trial applies a pragmatic randomized controlled design conducted under real-world clinical conditions. Integrating the VR intervention into standard care pathways without altering routine procedural workflows supports external validity. We acknowledge the lack of blinding as an inherent limitation of this intervention. However, to minimize bias, standardized outcome assessment procedures are applied uniformly across study groups. When interpreting future findings, such common trade-offs between internal control and clinical feasibility must be considered.

This approach is consistent with our hypothesis that immersive VR exerts its effects by shaping the emotional and experiential context of the procedure.

Our study uses a mixed-methods design, which pairs quantitative outcome measures with qualitative analyses, representing a methodological strength. The quantitative assessments integrate the patient’s self-reported anxiety and pain levels with objective physiological parameters, while the qualitative evaluations provide insight into the patient’s comprehensive understanding and experience of the entire procedure. Because immersive technologies often exert subtle or context-dependent effects, such effects may not be fully captured by quantitative measures alone. Therefore, the qualitative assessment will contribute to enriching the interpretation of the trial outcomes and to supporting a better understanding of the patient-reported experience. Additionally, in oncology settings, where patient profiles and treatment histories may influence procedural experience, the study prespecifies several factors that may influence procedural experience, including baseline anxiety, pharmacological therapies, and clinical context; all of this information is collected in advance. Acknowledging these potential confounders enhances methodological transparency and allows for a more accurate interpretation of outcome variability. Including all these considerations is essential to avoid oversimplified conclusions regarding intervention efficacy.

From a clinical perspective, this trial evaluates VR as a complementary, non-pharmacological intervention designed to enhance, not replace, established protocols. We conceptualize VR strictly as a supplement to standard care rather than a substitute for analgesic therapy or human interaction. Ultimately, if proven beneficial, VR offers a scalable, low-risk strategy to improve patient experience during routine oncological procedures.

Despite its methodological strengths, the trial presents anticipated limitations. Although the planned sample size is adequate for detecting moderate effects and assessing the primary exploratory objectives of the study, it may not be sufficient to identify subtle differences, particularly for secondary quantitative outcomes. However, the sampling strategy and planned sample size are appropriate for an exploratory, pragmatic, single-centre randomized trial and are sufficient to generate clinically meaningful estimates for the anxiety-driven component of the primary composite endpoint.

Moreover, while integrating both quantitative and qualitative criteria is a methodological strength, patient-reported parameters naturally increase statistical variability. This inherent variance can complicate the interpretation of outcomes. Furthermore, the single-centre design may limit generalizability, as institutional care pathways and patient characteristics may vary across oncology settings.

In addition, the pragmatic, unblinded design represents an inherent limitation. Given the reliance on patient-reported outcomes, expectation or performance bias cannot be excluded and should be considered when interpreting effect estimates. However, patient awareness of the intervention reflects real-world clinical practice and constitutes a meaningful component of the experiential effect of immersive VR.

Nevertheless, the consecutive recruitment of a real-world clinical population and full alignment with prospective trial registration support the internal validity of the findings and enhance their relevance to routine clinical practice. These aspects should be taken into account when interpreting the results and planning future confirmatory studies.

Future research may build upon the present study by extending recruitment to multiple centres, increasing sample size, and exploring subgroup effects among patients with higher baseline anxiety or limited prior procedural exposure. Longitudinal designs may also help clarify whether a positive procedural experience facilitated by VR influences longer-term outcomes, such as device acceptance, adherence to therapy, or willingness to undergo subsequent invasive procedures.

In conclusion, this protocol addresses a relevant clinical and technological gap by systematically evaluating immersive VR in the context of PICC and PICC-PORT placement, with a primary focus on a multidimensional patient-reported procedural experience within a non-aggregated composite framework. The findings of this trial are expected to contribute meaningful evidence to the growing field of digital health and to inform the development of more human-centred approaches to procedural care in oncology.

## Data Availability

The data supporting this study protocol are available from the corresponding author upon reasonable request. The data are not publicly available due to privacy and ethical restrictions.

## Supplementary Materials

The following supporting information can be downloaded at: https://www.mdpi.com/article/10.3390/nursrep16050165/s1, Supplementary File S1: Technical specifications of the virtual reality headset used in the study. Supplementary File S2: Approved data set.

## Public Involvement Statement

No public involvement in any aspect of this research.

## Guidelines and Standards Statement

This manuscript was drafted in accordance with the SPIRIT 2025 (Standard Protocol Items: Recommendations for Interventional Trials) checklist for randomized trial protocols to ensure comprehensive and transparent reporting. A complete list of reporting guidelines is available via the EQUATOR Network (https://www.equator-network.org/).

## References

[1] Faruki A., Nguyen T., Proeschel S., Levy N., Yu J., Ip V., Mueller A., Banner-Goodspeed V., O’Gara B. (2019). Virtual reality as an adjunct to anesthesia in the operating room. Trials.

[2] Ahmadpour N., Randall H., Choksi H., Gao A., Vaughan C., Poronnik P. (2019). Virtual reality interventions for acute and chronic pain management. Int. J. Biochem. Cell Biol..

[3] Lassen K.L., Hermander K., Jildenstål P., Wagner N., Augustinsson A., Sjöberg C., Geisler A. (2025). Virtual reality is safe and can reduce in-hospital anxiety and pain: A systematic review with meta-analyses and trial sequence analyses. Eur. J. Pain.

[4] Ioannou A., Papastavrou E., Avraamides M.N., Charalambous A. (2020). Virtual reality and symptom management of anxiety, depression, fatigue, and pain: A systematic review. SAGE Open Nurs..

[5] Oh H., Son W. (2022). Cybersickness and its severity arising from virtual reality content. Sensors.

[6] Simón-Vicente L., Rodríguez-Cano S., Delgado-Benito V., Ausín-Villaverde V., Delgado E.C. (2024). Cybersickness: A systematic literature review of adverse effects related to virtual reality. Neurología (Engl. Ed.).

[7] Addab S., Hamdy R., Thorstad K., Le May S., Tsimicalis A. (2022). Use of virtual reality in managing paediatric procedural pain and anxiety: An integrative literature review. J. Clin. Nurs..

[8] Kreidieh F.Y., Moukadem H.A., Saghir N.S.E. (2016). Overview, prevention and management of chemotherapy extravasation. World J. Clin. Oncol..

[9] AIOM Working Group Nursing (2021). Gestione Infermieristica Degli Accessi Vascolari Centrali a Medio e Lungo Termine nel Paziente Oncologico [Nursing Management of Medium- and Long-Term Central Venous Access Devices in Oncology Patients].

[10] Brescia F., Pittiruti M., Spencer T.R., Dawson R.B. (2024). The SIP protocol update: Eight strategies, incorporating Rapid Peripheral Vein Assessment (RaPeVA), to minimize complications associated with peripherally inserted central catheter insertion. J. Vasc. Access..

[11] Brescia F., Annetta M.G., Pinelli F., Pittiruti M.A. (2024). GAVeCeLT bundle for PICC-PORT insertion: The SIP-Port protocol. J. Vasc. Access..

[12] Ivziku D., Gualandi R., Pesce F., De Benedictis A., Tartaglini D. (2022). Adult oncology patients’ experiences of living with a central venous catheter: A systematic review and meta-synthesis. Support Care Cancer.

[13] Nicholson J., Davies L. (2013). Patients’ experiences of the PICC insertion procedure. Br. J. Nurs..

[14] Serin Z.S., Kazan E.E. (2025). The effect of using thermomechanical stimulation and virtual reality glasses during peripheral intravenous catheterization on pain and patient satisfaction: A randomized controlled trial. J. Emerg. Nurs..

[15] Schaake R., Leopold I., Sandberg A., Zenk B., Shafer L., Yu D., Lu X., Theingi S., Udongwo A., Cohen G.S. (2024). Virtual reality for the management of pain and anxiety for interventional radiology procedures: A prospective, randomized pilot study on digital sedation. J. Vasc. Interv. Radiol..

[16] Sargut M., Novotny A., Friess H., Kranzfelder M. (2025). Virtual reality in surgery: Minimizing stress and pain in patients undergoing minor-surgical procedures under local anesthesia—Results of a feasibility study. Int. J. Comput. Assist. Radiol. Surg..

[17] Steinkraus K.C., Feldmann H., Hunold L.S., Graf S., Dörr-Harim C., Nasir N., Michalski C.W., Hüttner F.J. (2024). Impact of virtual reality hypnosedation on perioperative pain and anxiety in port implantation under local anesthesia: A randomized controlled pilot trial (VIP Trial). Perioper. Med..

[18] Galinha-de-Sá F., Velez M. (2022). Van Kaam’s phenomenology: Theoretical-methodological contributions to nursing research. Rev. Gauch. Enferm..

[19] Chan A.-W., Boutron I., Hopewell S., Moher D., Schulz K.F., Collins G.S., Tunn R., Aggarwal R., Berkwits M., A Berlin J. (2025). SPIRIT 2025 statement: Updated guideline for protocols of randomised trials. BMJ.

[20] Gray R., Bressington D., Mackay B., Jones M., Thompson D.R. (2025). Perils of precisely equal group size in randomised controlled trials. Nurs. Rep..

[21] European Parliament, Council of the European Union (2017). Regulation (EU) 2017/745 on Medical Devices [Internet]. Off. J. Eur. Union.

[22] Hopewell S., Chan A.W., Collins G.S., Hróbjartsson A., Moher D., Schulz K.F., Tunn R., Aggarwal R., Berkwits M., Berlin J.A. (2025). CONSORT 2025 statement: Updated guideline for reporting randomised trials. Lancet.

[23] Lincoln Y.S., Guba E.G. (1985). Naturalistic Inquiry.

