# Supplementary material for: Design and Protocol of a Randomised Controlled Trial Evaluating Virtual Reality to Improve Patient Experience During PICC and PICC-PORT Placement in Oncology Patients
Source: Nurs Rep. 2026 May 13;16(5):165. doi: 10.3390/nursrep16050165 (PMC13209644; doi:10.3390/nursrep16050165)
Supplement: Supplementary file 1 [file nursrep-16-00165-s001.zip › nursrep-4245899-supplementary.pdf]

SUPPLEMENTARY MATERIALS

Supplementary File S1 – Technical specifications of the HypnoVR system, the virtual reality headset used in the study.

The technical description is based on manufacturer documentation and regulatory materials.

The HypnoVR system is a virtual reality–based medical device designed to support the management of pain, stress, and anxiety in clinical settings. It provides immersive hypnotic experiences intended to be used as an adjunct or alternative to pharmacological interventions.

HypnoVR is classified as a Class I medical device in accordance with Regulation (EU) 2017/745. The system is non-invasive and software-based, and it does not incorporate medicinal substances, biological materials, or implantable components.

The HypnoVR solution consists of two integrated software components: the HypnoVR application and the Compagnon software. These components are designed to be used together during clinical procedures, enabling healthcare professionals to deliver standardized immersive sessions to patients.

The intervention is delivered through a virtual reality headset, providing immersive audiovisual environments combined with guided hypnosis protocols. The system uses synchronized visual and auditory stimuli to promote distraction and cognitive modulation, with the aim of reducing the perception of pain, stress, and anxiety during medical procedures.

The content delivered through the system is standardized and reproducible across sessions, ensuring consistency in the administration of the intervention. The solution is intended for use in controlled clinical environments and can be integrated into routine care workflows as a supportive non-pharmacological intervention.

From a safety perspective, the software does not require specific storage conditions and does not present risks associated with sterility or implantation, as it is entirely digital. Standard precautions include adherence to the user manual and appropriate use by trained healthcare professionals.

HypnoVR is developed and manufactured by HypnoVR SAS (Lampertheim, France), and complies with applicable European regulatory requirements for medical devices.

Supplementary File S2: Approved data set (Data Collection Form)

**Allocation**		
Timing	Variable	Response Options
Pre-procedure	Intervention arm	VR+SOC / SOC
**Socio-demographic Data**		
Timing	Variable	Response Options
Pre-procedure	Sex	Female / Male
	Age	Numeric
	Education	Primary / Lower secondary / Upper secondary / University
Clinical Data		
Timing	Variable	Response Options
Pre-procedure	Oncological disease type	Open text
	Disease stage	Staging / Active treatment / Palliative
	Antiarrhythmic therapy	Yes/No
	Antihypertensive therapy	Yes/No
	Referring unit	Medical / Surgical / Day-unit / Hospice / ICU
	Time request-procedure	Same day / 1 day / 2–7 days / 8–15 / >15
	Main indication	Oncological therapy / Blood sampling / Parenteral nutrition
	First PICC/PICC-PORT?	Yes/No
	Previous device	PICC / PICC-PORT / PORT / CVC / Groshong / Other / NA
	Device placed	PICC / PICC-PORT
Vital signs baseline		
Timing	Variable	Method
Pre-procedure	HR – BP – Pain	VAS 1–10
	Pain location	Open text
Baseline anxiety		

Timing	Variable	Method
Pre-procedure	Anxiety VAS-A	1–10
	Factors contributing	Open text
Post-procedure vital signs		
Timing	Variable	Method
Post-procedure	HR – BP – Pain	VAS 1–10
	Pain location	Open text
Post-procedure anxiety		
Timing	Variable	Method
Post-procedure	Anxiety VAS-A	1–10
	Impact of care on anxiety	Likert 1–4
Device insertion data		
Timing	Variable	Method
Post-procedure	Number of punctures	Numeric
	Procedure duration	Minutes
	Local anesthetic	Ropi / Lido / Mepi / Other
	Anesthetic dose	Numeric
**Procedural setting**		
Timing	Variable	Method
Post-procedure	Room evaluation	Likert 1–4 + Open text
**Comfort**		
Timing	Variable	Method
Post-procedure	Comfort during procedure	Likert 1–4 + Open text
Expectations		
Timing	Variable	Method
Post-procedure	Was procedure as expected?	Likert 1–4 + Open text
	How different?	Open text
**Overall experience VR+SOC**		
Timing	Variable	Method
Post-procedure	VR video pleasant? distraction?	Likert 1–4 + Open text

**Overall experience SOC**		
Timing	Variable	Method
Post-procedure	Emotional impact of procedure	Likert 1–4 + Open text
Recommendation		
Timing	Variable	Method
Post-procedure	Would recommend centre?	Likert 1–4 + Open text
**Improvement suggestions**		
Timing	Variable	Method
Post-procedure	Suggestions for improvement	Open text
